# A Systematic Literature Review of the Natural History of Respiratory, Swallowing, Feeding, and Speech Functions in Spinal Muscular Atrophy (SMA)

**DOI:** 10.3233/JND-230248

**Published:** 2024-09-03

**Authors:** Yasmina Martí, Valerie Aponte Ribero, Sarah Batson, Stephen Mitchell, Ksenija Gorni, Nicole Gusset, Maryam Oskoui, Laurent Servais, Nicolas Deconinck, Katlyn Elizabeth McGrattan, Eugenio Mercuri, C. Simone Sutherland

**Affiliations:** a F. Hoffmann-La Roche Ltd, Basel, Switzerland; b Mtech Access Limited, Bicester, UK; c SMA Europe, Freiburg, Germany; d SMA Schweiz, Heimberg, Switzerland; e Departments of Pediatrics and Neurology Neurosurgery, McGill University, Montreal, Canada; f MDUK Oxford Neuromuscular Centre & NIHR Oxford Biomedical Research Centre, University of Oxford, Oxford, UK; g Department of Pediatrics, Division of Child Neurology, Centre de Références des Maladies Neuromusculaires, University Hospital Liège & University of Liège, Liège, Belgium; h Neuromuscular Reference Center, UZ Gent, Ghent, Belgium; i Department Paediatric Neurology, Centre de Références des Maladies Neuromusculaires, Hôpital Universitaire des Enfants Reine Fabiola (HUDERF), Hôpital Universitaire de Bruxelles, Université Libre de Bruxelles, Brussels, Belgium; j Department of Speech-Language-Hearing Science, University of Minnesota, Minneapolis, MN, USA; k Department of Rehabilitation, Masonic Children’s Hospital, Minneapolis, MN, USA; l Pediatric Neurology Institute, Catholic University and Nemo Pediatrico, Fondazione Policlinico Gemelli IRCCS, Rome, Italy; m Centro Clinico Nemo, Fondazione Policlinico Gemelli, IRCCS, Rome, Italy

**Keywords:** Spinal muscular atrophy, neuromuscular diseases, rare diseases, natural history, respiratory function tests, speech, deglutition, review

## Abstract

**Background:**

Respiratory and bulbar dysfunctions (including swallowing, feeding, and speech functions) are key symptoms of spinal muscular atrophy (SMA), especially in its most severe forms. Demonstrating the long-term efficacy of disease-modifying therapies (DMTs) necessitates an understanding of SMA natural history.

**Objective:**

This study summarizes published natural history data on respiratory, swallowing, feeding, and speech functions in patients with SMA not receiving DMTs.

**Methods:**

Electronic databases (Embase, MEDLINE, and Evidence-Based Medicine Reviews) were searched from database inception to June 27, 2022, for studies reporting data on respiratory and/or bulbar function outcomes in Types 1–3 SMA. Data were extracted into a predefined template and a descriptive summary of these data was provided.

**Results:**

Ninety-one publications were included: 43 reported data on respiratory, swallowing, feeding, and/or speech function outcomes. Data highlighted early loss of respiratory function for patients with Type 1 SMA, with ventilatory support typically required by 12 months of age. Patients with Type 2 or 3 SMA were at risk of losing respiratory function over time, with ventilatory support initiated between the first and fifth decades of life. Swallowing and feeding difficulties, including choking, chewing problems, and aspiration, were reported in patients across the SMA spectrum. Swallowing and feeding difficulties, and a need for non-oral nutritional support, were reported before 1 year of age in Type 1 SMA, and before 10 years of age in Type 2 SMA. Limited data relating to other bulbar functions were collated.

**Conclusions:**

Natural history data demonstrate that untreated patients with SMA experience respiratory and bulbar function deterioration, with a more rapid decline associated with greater disease severity. This study provides a comprehensive repository of natural history data on bulbar function in SMA, and it highlights that consistent assessment of outcomes in this area is necessary to benefit understanding and approval of new treatments.

## INTRODUCTION

Spinal muscular atrophy (SMA) is an autosomal recessive, progressive neuromuscular disease characterized by a deficiency in the survival of motor neuron (SMN) protein [1, 2], caused by loss-of-function mutations of or deletions within the *SMN1* gene. A paralogous gene, *SMN2*, produces low levels of SMN protein; however, these are insufficient to compensate for the lack of *SMN1* [1]. Although a rare disease, with an estimated incidence of 1 in 6,000 to 1 in 10,000 live births, SMA is the leading genetic cause of infant mortality in the absence of treatment [1]. SMA exists on a spectrum and has historically been classified into five types (Type 0–4), based on age at symptom onset and motor milestone achievement [3]. Patients with Type 1 SMA have disease onset at < 6 months of age, are never able to sit independently, and without treatment have a life expectancy of < 2 years. In Type 2 SMA, patients have disease onset at 6–18 months of age, can sit independently, but are never able to walk and have a life expectancy of 20–40 years. Patients with Type 3 SMA have symptom onset at > 18 months of age and have a normal life expectancy [1]. They are able to walk, but may lose this ability over time [3]. Type 1 SMA is the most common form of the disease, accounting for approximately 50–80% of SMA diagnoses [2, 4]. The severity of SMA is generally inversely correlated to *SMN2* copy number [2]; however, this is not absolute due to additional genetic and epigenetic disease modifiers [2, 5, 6].

If untreated, patients with SMA experience progressive muscle denervation, skeletal muscular atrophy, overall weakness, and motor function loss [2]. Degeneration of the motor neurons controlling breathing leads to respiratory muscle weakness, predominantly affecting the intercostal muscles [7]. Respiratory complications arising from this include breathing difficulties and loss of effective cough that lead to the need for ventilatory intervention and assistance with secretion clearance, which can negatively affect the prognosis [8, 9]. Degeneration of the motor neurons that control bulbar function contributes to bulbar muscle weakness, which manifests as swallowing difficulties (i.e., abnormalities in the swallowing mechanism) and feeding difficulties (i.e., problems with eating activities, such as eating with a spoon or chewing) [10]. These can be exacerbated by abnormal head postures caused by scoliosis or scoliosis surgery [11]. Feeding support (e.g., nasogastric tube [NGT] or gastrostomy) may be required to enable adequate nutrition and prevent choking, aspiration, and respiratory infections [12–14]. Bulbar muscle weakness also affects the muscles involved in airway protection and causes intelligibility problems [9, 15–17]. Therefore, the impact of SMA on respiratory and bulbar functions can affect patient morbidity, quality of life, and healthcare resource utilization [8, 9, 13–15, 18].

Deficits in the respiratory and bulbar functions have been previously reported in patients with SMA [8, 9, 13–15, 19–21]. However, while disease-modifying therapies (DMTs) have been proven to have beneficial effects on survival and motor function [22], the lack of consistency in the assessment of respiratory and bulbar function has restricted the systematic evaluation of DMT effects on these outcomes [12, 15, 19, 20]. As adoption of DMTs becomes more widespread, the ability to prospectively gather SMA natural history data, which are often the appropriate comparator in the evaluation of treatment responses [23–25], is becoming increasingly difficult. Therefore, existing patient-level data that thoroughly describe SMA natural history across relevant clinical outcomes and the clinical spectrum of disease are needed.

The objective of this systematic literature review (SLR) was to summarize published data on respiratory and bulbar functions, including swallowing, feeding, and speech, in patients with SMA who were not receiving DMTs.

## METHODS

### Standard protocol approvals and registrations

The Preferred Reporting Items for Systematic reviews and Meta-Analyses for Protocols 2020 guidelines [26] were used to report this SLR. The predefined protocol for the SLR was not registered. Ethical approval was not required as data used in our analyses were extracted from published studies.

### Search methodology

Searches were conducted on May 29, 2021, and updated on June 27, 2022. The electronic databases used for the systematic search were Embase, MEDLINE (including MEDLINE Epub Ahead of Print, MEDLINE In-Process and Other Non-Indexed Citations, and MEDLINE Daily), and Evidence-Based Medicine Reviews (incorporating: the Health Technology Assessment Database, National Health Service Economic Evaluation Database, Cochrane Central Register of Controlled Trials, Database of Abstracts of Reviews of Effects, and the Cochrane Database of Systematic Reviews), accessed via the Ovid platform. A full list of search terms is available in Supplementary Tables 1, 2, and 3. Additional searches were also performed to identify relevant publications published as conference proceedings (2018–2022), as citations in included publications, and on health technology assessment body websites.

The search strategy for the systematic search was previously published in Aponte Ribero et al. [27].

Eligibility criteria were determined based on the Population, Intervention, Comparison, Outcomes, and Study (PICOS) framework [28]. The aim of these analyses was to summarize the data available on respiratory and bulbar outcomes in SMA. Therefore, we framed our eligibility criteria to identify studies reporting data on respiratory function, the need for ventilatory support or tracheostomy, and bulbar function (in particular, swallowing, feeding, and speech) in patients with Types 1–3 SMA, with a primary interest in time-to-event data (Supplementary Table 4). Eligible studies included observational studies, case–control studies, cross-sectional studies, and case series.

### Study selection and data extraction

Screening of citations by title and abstract (all citations) and full publication (for citations deemed potentially relevant based on title and abstract screening) was conducted by two independent analysts (SB and SM). Data on study design, baseline characteristics, and treatment outcomes were extracted into summary tables by an analyst (SB). All data were independently checked against the source document by a second analyst (SM), and any disputes resolved by consensus.

### Assessment of bias and quality of evidence

Risk of bias assessments based on the study design of the individual studies were conducted by an analyst (SB) using the analytical cross-sectional study checklist from the Joanna Briggs Institute [29].

### Data synthesis and inclusion of studies for analysis

Although the search covered several functional outcomes, for the purpose of this analysis, only studies reporting outcomes on respiratory and bulbar functions (including terms relating to swallowing, feeding, and speech) were considered. Natural history data on motor function, scoliosis, and contracture outcomes were previously published by Aponte Ribero et al. [27].

Numerical time-to-event data (i.e., mean or median time-to-event) and non-time-to-event data (i.e., rates, mean baseline, follow-up, or change from baseline score) for the relevant outcomes were extracted and summarized.

## RESULTS

**Fig. 1:**
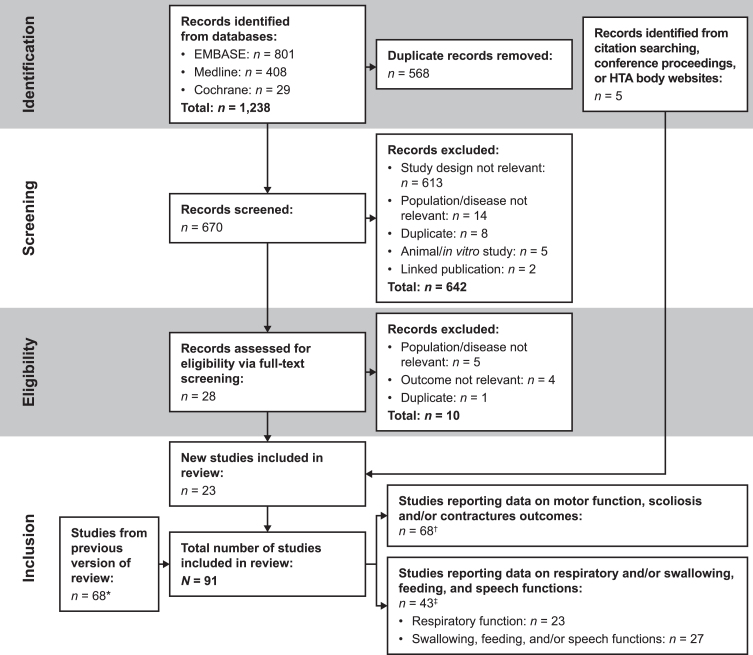
PRISMA flow diagram. Figure modified from Aponte Ribero et al. 2023 [27]. *A PRISMA flow diagram of the original search is provided in Supplementary Figure 1. ^†^Studies that only reported on motor function, scoliosis, and/or contracture outcomes were not considered further in this manuscript; data from these studies were described separately in Aponte Ribero et al. 2023. ^‡^Some of the 43 studies reported on both respiratory and bulbar function outcomes. Abbreviations: HTA = health technology assessment; PRISMA = Preferred Reporting Items for Systematic reviews and Meta-Analyses.

In the original search (May 29, 2021), 6,475 articles were identified (Supplementary Figure 1). Out of these, 5,027 titles and abstracts were screened, of which 91 articles were selected as potentially relevant. Of these, 26 articles were excluded based on the Population, Interventions, Comparators, and Outcomes (PICO) framework criteria. Sixty-eight articles were selected for inclusion in the SLR (including three articles identified via handsearching). In the updated search (June 27, 2022), an additional 23 records were deemed eligible for inclusion. In total, 91 publications were finally included in the SLR (Fig. 1); 43 of these reported data on respiratory, swallowing, feeding, and speech functions, which are considered in the present publication (Supplementary Table 5). The studies excluded from the SLR are indicated in Supplementary Table 6.

The reviewed studies covered registries across Europe, Asia, the USA, and Canada. Publication dates ranged from 1989–2022, with approximately 90% of studies published in 2012 or later. Studies included patients with Types 0–4 SMA. Only one study reported by *SMN2* copy number rather than SMA type [30]. There was limited reporting of patient characteristics and variability in the definitions of SMA types, SMA phenotypes, and patient characteristics. In addition, variability in the definitions for swallowing and feeding was noted across the reviewed studies, with some of these referring to swallowing difficulties as feeding difficulties.

A total of 23 publications reported data relating to respiratory function, including numerical time-to-event data for respiratory failure/support (Table 1) [8, 13, 14, 31], as well as Kaplan–Meier (KM) [8, 9, 14, 32, 33] data, and additional cross-sectional and longitudinal data for forced vital capacity (FVC) (Supplementary Table 7), peak cough flow (PCF) (Table 2), sniff nasal inspiratory pressure (SNIP) (Table 3), and polysomnography data (Table 4) [7, 8, 13, 14, 31–48]. The SLR identified 27 publications that reported data relating to swallowing, feeding, and/or speech functions in patients with SMA (Supplementary Data Extraction Table).

**Table 1:** Summary of time-to-event data for respiratory failure/support

Author year	Data source, territory, population	N	Age	Outcomes
Aguerre 2020 [31]	A third-level pediatric hospital in Argentina	59	Children	Mean age at developing respiratory failure (SD), months
	Type 1 SMA			•Type 1b SMA: 5.9 (2.32)
				•Type 1c SMA: 13.8 (5.6)
Kaneko 2017 [14]	Japanese individuals enrolled by questionnaire who completed the questionnaire	112	Adults and children	Median age at start of non-invasive positive pressure ventilation (range), months:
	Types 1–3 SMA	•Type 1 : 47		•Type 1a SMA (*n* = 3): 14 (5–51)
		•Type 2 : 42		•Type 1b SMA (*n* = 2): 23 (15–29)
		•Type 3 : 23		•Type 2a SMA (*n* = 6): 105.5 (27–379)
				•Type 2b SMA (*n* = 16): 37 (24–160)
				•Type 3a SMA (*n* = 2): 336 and 468 (two patients)
				•Type 3b SMA (*n* = 1): 600 (single patient)
				Median age at start of TPPV (range), months:
				•Type 1a SMA (*n* = 35), 6 (2–51)
				•Type 1b SMA (*n* = 3), 12 (11–122)
Wadman 2021 [13]	Data from a single-center retrospective study in the UK and a prospective multicenter study in Italy	146	Children and adolescents	Median age at start of NIV (range), years
	Type 2 SMA			•Total cohort (*n* = 146): 4 (1–17)
				•UK (*n* = 72): 6 (2–17)
				•Italy (*n* = 74): 3 (1–11)
Wadman 2017 [8]	Dutch patient organization for neuromuscular diseases	200	Children and adolescents	Mean age at start of NIV (range), years:
	The Netherlands Types 0–4 SMA	•Type 0/1a: 3		•Type 0/1a SMA (*n* = 3): 0
		•Type 1b: 16		•Type 1b SMA (*n* = 16): 0.8 (0.6–1.1)
		•Type 1c: 23		•Type 1c SMA (*n* = 23): 11 (1.3–36)
		•Type 2a: 49		Mean age at start of ventilatory support at night (range), years:
		•Type 2b: 38		•Type 2a SMA (*n* = 49): 14 (2–38)
		•Type 3a: 33		•Type 2b SMA (*n* = 38): 19 (2–66)
		•Type 3b: 33		•Type 3a SMA (*n* = 33): 46 (15–61.5)
		•Type 4 : 5		•Type 3b SMA (*n* = 33): 40 (39.6)
				Mean age at start of ventilatory support > 16 hours/day (range), years:
				•Type 2a SMA (*n* = 49): 20 (16–24)
				•Type 2b SMA (*n* = 38): 31 (31)
				•Type 3a SMA (*n* = 33): 34 (20–47)
				•Type 3b SMA (*n* = 33) 0

Abbreviations: NIV = non-invasive ventilation; SD = standard deviation; SMA = spinal muscular atrophy; SMN = survival of motor neuron; TPPV = tracheostomy positive pressure ventilation.

**Table 2:** Summary of studies reporting PCF outcomes

Author year	Data source, territory, population	N	Age	PCF outcome data reported
Chabanon 2018 [44]	NatHis-SMA	81	Adults and children	**PCF % predicted values, median (IQR):**
	Belgium, France, and Germany			•Type 2 SMA, non-sitter (*n* = 15): 43 (34–62)
	Types 2 and 3 SMA			•Type 2 SMA, sitter (*n* = 9): 68 (51–79)
				•Type 3 SMA, non-ambulant (*n* = 9): 88 (67–112)
				•Type 3 SMA, ambulant (*n* = 10): 79 (70–88)
				•Overall (*n* = 43): 69 (45–84)
				Patients > 6 years of age performed PCF assessments in the sitting position which were captured with the Vitalograph spirometer; the best results of three measurements were selected for analysis
Kapur 2019 [42]	Children attending the Children’s Health Queensland, Brisbane, Australia Types 1–3 SMA	25	Children	**PCF, median L/min (IQR) from a cross-sectional study:**
				•Type 2 SMA: 178.8 (246.0)
				•Type 3 SMA: 277.8 (124.8)
				•All patients: 231.9 (181.8)
				Median Z scores lower in patients who needed NIV (results displayed graphically); *p* = 0.75
				Children who refused to perform a respiratory test were not forced. Standard testing methods were used
Veldhoen 2022 [7]	Patients enrolled in this study were participating in a prospective, population-based prevalence cohort study on SMA in the Netherlands (Wadman 2017, 2018, 2020) Types 1–3 SMA	80	Adults and children	**Longitudinal patterns of PCF reported graphically**
				•PCF was lowest in Type 1c SMA:<160 L/min throughout life
				•Patients with Type 2 SMA reached 160–270 L/min, with clear differences between Types 2a and 2b SMA
				•Patients with Type 3 SMA had higher PCF values from earlier ages onwards in comparison with Type 2b SMA, but median values were still below normal

Abbreviations: IQR = interquartile range; NatHis = natural history; NIV = non-invasive ventilation; PCF = peak cough flow; SMA = spinal muscular atrophy.

**Table 3:** Summary of studies reporting SNIP outcomes

Author year	Data source, territory, population	N	Age	SNIP outcome data reported
Chabanon 2018 [44]	NatHis-SMA	81	Adults and children	SNIP % predicted values, median (IQR)
	Belgium, France, and Germany			•Type 2 SMA, non-sitter (*n* = 15): 35 (22–64)
	Types 2 and 3 SMA			•Type 2 SMA, sitter (*n* = 9): 33 (26–56)
				•Type 3 SMA, non-ambulant (*n* = 9): 56 (27–99)
				•Type 3 SMA, ambulant (*n* = 10): 45 (35–76)
				•Overall (*n* = 43): 39 (28–60)
				Patients > 6 years of age performed SNIP assessments in the sitting position which were captured with the MicroRPM device; the best results of three measurements were selected for analysis
Kapur 2019 [42]	Children attending the Children’s Health Queensland, Brisbane, Australia	25	Children	**SNIP median Z score (IQR) (*n*** = **14) from a cross-sectional study**
	Types 1–3 SMA			•Type 2 SMA: –2.53 (2.61)
				•Type 3 SMA: –1.32 (2.47)
				•All patients: –2.06 (2.87)
				Median Z scores were lower in patients who needed NIV (results displayed graphically); *p* = 0.25
				Children who refused to perform a respiratory test were not forced. Standard testing methods were used
Veldhoen 2022 [7]	Patients enrolled in this study were participating in a prospective, population-based prevalence cohort study on SMA in the Netherlands (Wadman 2017, 2018, 2020)	57	Adults and children	**SNIP, median (cmH_**2**_O):**
	Types 1–3 SMA			•Type 1c SMA: 33
				•Type 2a SMA: 44
				•Type 2b SMA: 59
				•Type 3a SMA: 58
				•Type 3b SMA: 55
				Results also reported graphically in box plots
				There was a significant trend of increasing SNIP with less severe SMA types (*p* = 0.0053)
				All SNIP outcomes were below 75 cmH_2_O, which is considered the lower limit of normal

Abbreviations: IQR = interquartile range; NatHis = natural history; NIV = non-invasive ventilation; SMA = spinal muscular atrophy; SNIP = sniff nasal inspiratory pressure.

**Table 4:** Summary of studies reporting polysomnography outcomes

Author year	Data source, territory, population	N	Age	Polysomnography outcome data reported
Kapur 2019 [42]	Children attending the Children’s Health Queensland, Brisbane, Australia	25	Children	Median total AHI (IQR) from a cross-sectional study
	Types 1–3 SMA			•Type 1 SMA: 7.6 (4.3)
				•Type 2 SMA: 2.9 (2.4)
				•Type 3 SMA: 3.1 (3.5)
				•All patients: 3.1 (4.9)
				Median REM AHI (IQR) from a cross-sectional study
				•Type 1 SMA: 20.0 (45.7)
				•Type 2 SMA: 5.8 (9.9)
				•Type 3 SMA: 4.6 (6.4)
				•All patients: 5.8 (9.1)
				Full diagnostic polysomnography was performed by standard methods

Abbreviations: AHI = apnea–hypopnea index; IQR = interquartile range; REM = rapid eye movement; SMA = spinal muscular atrophy.

### Respiratory function outcomes

#### Requirement for ventilatory support or time to respiratory failure

Details of the development of respiratory failure or the requirement for ventilatory support, in the form of tracheostomy positive pressure ventilation (TPPV) or non-invasive ventilation (NIV), were extracted in the form of KM curve data [8, 9, 14, 32, 33] or numerical time-to-event data (Table 1) [8, 13, 14, 31].

Overall, the reviewed studies demonstrated that patients with Type 1 SMA required ventilatory support by 12 months of age [8, 9, 14, 32]. Kaneko et al. 2017 [14] reported that in patients with Type 1 SMA, a lack of head control and earlier age at disease onset were associated with a more rapid decline in respiratory function.

KM analyses of the timing of the introduction of TPPV illustrated that ∼80% of participants with Type 1a SMA (no head control, age at diagnosis ≤2 weeks) required TPPV by 12 months of age compared with ∼10% of those with Type 1b SMA (head control, age at diagnosis by ≤3 months) [14]. The median age at the initiation of TPPV was 6 months for patients with Type 1a SMA and 12 months for those with Type 1b SMA [14]. In patients with Type 1 SMA in the Pediatric Neuromuscular Clinical Research Network study dataset, the median age at which participants were reported to reach the composite endpoint of death or requiring permanent ventilation (> 16 hours/day of NIV) was 13.5 months (interquartile range [IQR] 8.1–22.0) [9]. No significant difference was reported between the age at which the composite endpoint was met for patients with Type 1b versus 1c SMA (11.9 months [IQR 7.0–22.0] vs 13.6 months [IQR 8.8–20.1]; *p* = 0.58) [9]. However, differences were observed by *SMN2* copy number; patients with two S*MN2* copies reached the composite endpoint at a younger age (median age 10.5 months [IQR 8.1–13.6]) compared with those with three *SMN2* copies (median age not reached; 25^th^ percentile: 22.0 months) [9].

Where compared, patients with Type 1 SMA typically required NIV at an earlier age than those with Type 2 or 3 SMA; the ages at which NIV was initiated for patients with Type 2 or 3 SMA were reported to span the first to fifth decades of life [8, 13, 14, 31]. In an analysis of patients with Type 2 or 3 SMA [8], patients with Type 2a SMA (patients who were able to sit independently, but never stood or walked) had the greatest risk of losing respiratory function over time. Based on KM analyses, the percentages of patients who were predicted to require nocturnal ventilatory support at 20 years of age were ∼45% for Type 2a SMA, ∼8% for Type 2b (patients who were able to sit independently and were able to stand or walk with support), ∼5% for Type 3a (symptom onset < 3 years), and none for Type 3b SMA (symptom onset > 3 years). At 25 years of age, ∼20% of patients with Type 2b SMA were predicted to need ventilatory support. At 50 years of age, ∼20% of patients with Type 3a SMA, and ∼10% of patients with Types 3b SMA (symptom onset > 3 years), were predicted to require ventilatory support [8].

A single study exploring breathing difficulties in patients with Type 2 SMA in the UK and Italy reported a lower median age at the start of NIV in participants in Italy versus the UK (3 vs 6 years), highlighting differences in clinical practice based on geographical location [13].

#### FVC

Data relating to FVC were reported in 16 studies (Supplementary Table 7) [33–48]. Where details of lung function tests were reported, assessments generally included spirometry, conducted with the patient in a sitting position, and the best results of three measurements were selected for analysis [33, 34, 39, 42, 44, 46]. The format of reporting of FVC data across the studies included baseline data (or data from cross-sectional studies) and, most commonly, longitudinal data which were generally reported as annual declines in predicted FVC across the studies (Supplementary Table 7). All the studies included patients with Type 2 and/or 3 SMA [33–48], while five studies additionally included patients with Type 1 and/or 4 SMA [35, 36, 39, 42, 48]. In general, increased disease severity corresponded with a lower FVC [35, 42, 44–46, 48].

Three studies reported longitudinal changes in FVC graphically and demonstrated that FVC is predicted to decline in a non-linear fashion over time in all SMA types [33, 39, 46]. These data indicated that decline in FVC is most pronounced at younger ages followed by a slower rate of decline (or even stable course) during adulthood in patients with Types 1–3 SMA. For example, Trucco et al. 2021 [33] reported that FVC % was predicted to decline by 4.2% per year between 5 and 13 years of age, followed by a decline of 1% per year after the age of 13 years in patients with Type 2 SMA. Similarly, in patients with Type 3 SMA, FVC was predicted to decline by 6.3% per year from 8–13 years of age, followed by a slower decline of 0.9% per year. A single study [33] reported the age at which clinically meaningful thresholds of predicted FVC (60, 40, and 20%) were achieved in patients with Types 2 and 3 SMA. The median age at which predicted FVC < 60% was met was 12.8 years for patients with Type 2 SMA. The median age was not reached across analyses of 40% and 20% predicted FVC for patients with Type 2 SMA or for any of the analyses in patients with Type 3 SMA.

#### PCF

PCF data were reported in three studies (Table 2) [7, 42, 44]. Chabanon et al. 2018 [44] reported in the baseline data of a prospective and longitudinal natural history study, that median PCF % predicted values were lowest (indicating more impaired lung function) in patients classified as Type 2 SMA non-sitters (43%) compared with Type 2 SMA sitters (68%) and those with Type 3 SMA (non-ambulant: 88%; ambulant: 79%). Similarly, in a cross-sectional study of childhood SMA, median PCF values (L/min) were lower in patients with Type 2 SMA than in patients with Type 3 SMA (178.8 vs 277.8 L/min; *p* = 0.23) [42]. In a natural history study that analyzed the longitudinal patterns of PCF in patients with Types 1–3b SMA [7], all but those with Type 3b SMA had a lowered PCF compared with the therapeutic thresholds of 270 L/min, indicating vulnerability to respiratory failure during otherwise trivial respiratory tract infections. PCF values were lowest in patients with Type 1c SMA (<160 L/min throughout life, the boundary below which secretion clearance becomes ineffective). Patients with Type 2 SMA reached values between 160 and 270 L/min after early childhood. For those with Type 2a SMA, median PCF remained around 160 L/min during adolescence and early adulthood, whereas in patients with Type 2b SMA, median PCF steadily increased until (early) adulthood. PCF values in patients with Type 3a SMA were higher from earlier ages onwards in comparison with Type 2b; however, median values were still well below therapeutic thresholds.

#### SNIP

Three studies reported data relating to SNIP, a summary of which is provided in Table 3 [7, 42, 44]. Although the studies reported different outcomes (SNIP % predicted values [44]; SNIP median Z scores [42]; SNIP in cmH_2_O [7]), there was a trend across studies of increasing SNIP with less severe SMA types. In the natural history study of patients with Types 1–3 SMA by Veldhoen et al. 2022 [7], the increase in SNIP values with less severe SMA was reported to be statistically significant (*p* = 0.0053). In addition, the SNIP results for all participants (Types 1c–3b) were below the lower limit of normal (75 cmH_2_O).

#### Polysomnography

A single study reported full diagnostic polysomnography results in patients with Types 1–3 SMA in terms of total apnea–hypopnea index (AHI) scores and rapid eye movement AHI scores (Table 4). The authors also compared diagnostic and NIV titration studies for children treated with NIV regardless of reason for NIV; an improvement in total AHI (NIV 3.65 vs no NIV 0.08; *p* < 0.04) and AHI during rapid eye movement sleep (NIV 12.9 vs no NIV 1.5; *p* < 0.05) was reported in participants requiring NIV [42].

### Swallowing, feeding, and speech function outcomes

Twenty six reviewed studies (i.e., all of the studies in Supplementary Data Extraction Table except Johnson et al. 2021) reported swallowing, feeding, and/or speech deficits that impeded the individuals’ ability to obtain full oral nutrition or to do so without experiencing symptoms, and one study was identified that reported intelligibility problems experienced by patients with SMA [16]. The prevalence of these disorders was noted to be impacted by SMA type and the extent of disease progression.

#### Swallowing and feeding difficulties

Thirteen studies reported on functional and physiologic characteristics of oropharyngeal swallowing difficulties [11, 13, 16, 33, 41, 44, 49–55]. These studies revealed a greater prevalence of difficulties with increasing SMA phenotype severity [11, 16, 33, 41, 44, 50, 55] and age [11].

The ability of patients with Type 1 SMA to eat by mouth declined with age: De Amicis et al. 2021 [54] reported that at a median age of 0.6 years, 83/105 patients (79%) were able to eat by mouth, whereas Choi et al. 2020 [51] reported that, among participants with a median age of 5.1 years (range 3.3–11 years), none (0/11) were able to eat. In a natural history study in infants with Type 1 SMA aged 3–24 months (*n* = 8), 75% obtained all nutrition by mouth [52]. However, 65% required suctioning to aid secretion management and 50% exhibited swallowing deficits (absent pharyngeal stripping wave, absent epiglottic inversion, and absent pharyngoesophageal segment opening) which limited bolus clearance from the pharynx (63%) and led to bolus airway entry (75%) [52].

In one assessment of bulbar function in 11 patients with Type 1 SMA (mean age 11.8 years [range 1–38]) [16], the majority were reported to have mastication problems (64%), fatigue associated with mastication (73%), swallowing difficulties (64%), choking more than once a day (82%), or coughing with solid foods (64%). Similarly, in an observational study of infants with Type 1 SMA receiving palliative care (*n* = 11, age at study inclusion 52 days [range 16–252]) [53]), the most frequently reported feeding difficulties included coughing when eating (91%); short nursing sessions without finishing feeds (72%); wet breathing during or after feeding (64%); frequent nursing sessions (55%); and sweating when feeding (55%).

The swallowing and feeding difficulties reported in patients with Type 2 or 3 SMA included problems bringing food to the mouth, chewing problems, jaw opening problems (affecting biting, laughing, and yawning), oral hypersensitivity, drooling, choking, aspiration, prolonged mealtime, weight loss, and dysphagia [11, 13, 16, 33, 41, 50]. Differences in outcome assessment across the reviewed studies limited the reporting of numerical trends in this SLR; however, it must be noted that swallowing difficulties and choking were the most frequent bulbar dysfunctions reported across the reviewed studies [13, 16, 33, 41, 50]. In children and adolescents with Type 2 SMA from the UK and Italy [13], the median age of onset of feeding difficulties was 6.5 years (range 0–16.5 years). Feeding difficulties were more frequent in Type 2a versus Type 2b SMA (*p* < 0.01), but the age at onset was not influenced by SMA subtype [13].

#### Requirement for non-oral feeding support

Data relating to the use of non-oral feeding support (NGT or gastrostomy tube) by patients with Type 1–3 SMA were collated from 18 publications [9, 13, 14, 17, 33, 35, 44, 51, 54, 56–64]. Findings from these investigations demonstrated that the proportion of patients with SMA who relied on non-oral feeding support increased with increasing phenotype severity [9, 14, 54, 57, 63]. Across these studies, it was reported that 0–100% [9, 14, 35, 51, 54, 56–58, 60], 0–50% [9, 14, 35, 54, 56, 57], and 0–4% [14, 35, 56, 57] of patients with Type 1, 2, and 3 SMA, respectively, relied on a feeding tube to meet nutritional needs. A sub-analysis of rates by SMA severity indicated that the proportion of patients with severe (corresponding to 1.1 on the Dubowitz’s decimal classification [87.5%; 7/8] or typical (1.5 on the Dubowitz’s decimal classification [75%; 6/8]) Type 1 SMA who required non-oral feeding support was greater than in those with a moderate phenotype (1.9 on the Dubowitz’s decimal classification [50%; 2/4]) over the 48-month assessmentperiod [58].

Three studies reported on the age that non-oral nutritional support was provided in patients with Type 1 and Type 2 SMA (Table 5) [9, 14, 51]. Patients with Type 1 SMA required non-oral nutritional support earlier than those with Type 2 SMA [9, 14, 51]. Age at the time of non-oral nutritional support placement ranged from 4–12.5 months for patients with Type 1 SMA [14, 51], and from 30–55 months in patients with Type 2 SMA [9].

**Table 5:** Summary of studies reporting time-to-event feeding support outcomes (n = 3)

Author year	Data source, territory, population	N	Age	Outcomes
Choi 2020 [51]	Retrospective chart reviews between January 2012 and March 2017 in a tertiary hospital Korea	11	Children	Median age months (IQR):
	Type 1 SMA			•Patients began tube feeding: 6 (3–7)
				•Discontinuation of oral breaks/milk feeding: 6 (3–6.5)
				The main reasons for switching to tube feeding were poor oral intake, recurrent pneumonia, and aspiration
Kaneko 2017 [14]	Japanese individuals enrolled by questionnaire who completed the questionnaire	112	Adults and children	Median age at the start of tube feeding support (range), months:
	Types 1–3 SMA	•Type 1 : 47		•Type 1a SMA (*n* = 36): 4 (1–40)
		•Type 2 : 42		•Type 1b SMA (*n* = 2): 12.5 (12–13)
		•Type 3 : 23		
Wadman 2021 [13]	Data from a single-center retrospective study in the UK and a prospective multicenter study in Italy	146	Children and adolescents	Median age at starting of feeding difficulties (range), years:
	Type 2 SMA			•Total cohort (*n* = 146): 6.5 (0–16.5)
				•UK (*n* = 72): 6.9 (0–17)
				•Italy (*n* = 74): 6.0 (2–10)
				Median age at starting nasogastric tube (range), years:
				•UK (*n* = 72): 4.1 (1–17)
				•Italy (*n* = 74): NA
				Median age at gastrostomy (range), years:
				•UK (*n* = 72): 7.3 (3–19)
				•Italy (*n* = 74): NA

Abbreviations: IQR = interquartile range; NA = not applicable; SMA = spinal muscular atrophy.

#### Speech difficulties

One study reported details of speech difficulties in patients with SMA (Types 1–3a) [16]. Speech difficulties included shortness of breath (46% in Type 1 SMA, 24% in Type 2 SMA, and 15% in Type 3a SMA) or fatigue (36% in Type 1 SMA, 16% in Type 2 SMA, and 15% in Type 3a SMA) associated with talking or being asked to repeat a conversation (46% in Type 1 SMA, 26% in Type 2 SMA, and 19% in Type 3a SMA), suffering from a sore throat (18% in Type 1 SMA, 20% in Type 2 SMA, and 26% in Type 3a SMA), or weak voice (27% in Type 1 SMA, 35% in Type 2 SMA, and 23% in Type 3 SMA). As reflected by these data, frequency of all speech difficulties increased with SMA severity except for sore throat and weak voice.

## DISCUSSION

DMTs are being increasingly introduced into the treatment pathway for SMA worldwide, making natural history comparator data essential to demonstrate the impact of treatment on long-term outcomes [25]. In line with the known SMA phenotypes, data collated in this SLR provided evidence that patients with Type 1 SMA experience rapid respiratory deterioration in the first year of life, typically requiring therapies to support airway clearance and ventilation. Among patients with Type 1 SMA, the requirement for ventilatory support was associated with an earlier age of disease onset, lack of head control, and lower *SMN2* copy number [9, 14]. Declines in respiratory function over time were also reported in patients with Type 2 or 3 SMA, with patients with Type 2a SMA being at a greater risk [8]. This review shows that patients with Type 2 or 3 SMA continue to be at risk of a decline in respiratory function even at older ages, with ventilatory support initiated between the first and fifth decades of life [8, 13, 14].

The progressive decline in motor function in SMA was reported to cause swallowing and feeding difficulties, and/or the requirement for nutritional support. Swallowing and feeding difficulties affected patients across the SMA spectrum (Types 1–3 SMA), and included chewing problems, choking, and aspiration [13, 14, 33, 41, 51]. A higher rate of feeding difficulties or the requirement for nutritional support, in the form of an NGT or gastrostomy placement, was associated with increasing SMA severity [14, 33, 41, 44, 51]. In fact, the requirement for an NGT or gastrostomy was consistently reported in patients with Type 1 SMA across studies, whereas few or no patients with Type 2 or 3 SMA were reported to receive this level of nutritional support [9, 35, 51, 54, 56–58, 60]. Speech and communication difficulties were additional aspects of bulbar function reported to be negatively affected by SMA [16].

This review highlights the limited data available in the literature on respiratory and bulbar function outcomes. Data for feeding difficulties, the need for nutritional support, and intelligibility problems were reported at baseline only, with no KM data or long-term follow-up data reported. Although information relating to a wide range of respiratory function outcomes was collated, except for predicted FVC, none or very limited data were identified for PCF, SNIP, polysomnography, oximetry, or blood gas analysis outcomes. In addition, few studies reporting KM, time-to-event, or long-term follow-up data on respiratory function in patients with SMA were identified.

Although the overall trend was that respiratory function declined over time in patients with SMA, where longitudinal predicted FVC data were reported, results should be interpreted with consideration of the impact of modeling choice, as FVC is predicted to decline in a non-linear fashion in all SMA types, with larger declines observed at younger ages, followed by stabilization at older ages. It is also important to note that spirometry requires patient cooperation to perform reliably. Spirometry may be difficult, especially in younger children who may not understand instructions, and in patients with severe muscle weakness who are unable to physically generate sufficient effort upon exhalation [65, 66], further highlighting the need to interpret these results with caution. FVC data were reported in children from 5 years of age; however, only one study [33] reported data from patients aged < 6 years (Supplementray Table 7). It is considered that reliable spirometry can generally be performed on children from 6 years of age; however, younger children are able to perform spirometry with training and normal reference ranges for spirometry are available from 4 years of age [68].

Potential differences in standard of care between countries should be considered for the interpretation of data on respiratory and nutritional interventions in patients with SMA, particularly in less severe SMA phenotypes where guidelines are less stringent, and where interventions depend on the evaluation of the treating physician [13].

In the interpretation of our results, it must be noted that definitions and outcome assessments varied across the reviewed studies. Most studies assessed measures of feeding/nutrition with a self-designed questionnaire and/or through clinical observations; one study used the Oral and Swallowing Abilities Tool checklist [49], a second used videofluoroscopic swallow study (VFSS) [52], and a third used a combination of a VFSS and the Neuromuscular disease Swallowing Status Scale [51]. A VFSS is recommended in SMA standard of care if clinical signs of dysphagia are present [69]. In addition, although subjective symptoms relating to the consequences of weak swallowing function (e.g., NGT feeding, gastrostomy placement, aspiration) were captured, reporting on objective measures of swallowing function was inconsistent across the reviewed studies.

As a limitation of this study, the systematic search was restricted to selected respiratory, swallowing, feeding, and speech functions, and as a result did not capture data relating to other signs of bulbar dysfunction such as drooling. In addition, although eligible publications were restricted to Types 1–3 SMA, there was inter-study heterogeneity in the definition of SMA phenotype and patient characteristics. Therefore, statistical comparisons were not possible, and only descriptive results were reported. Nevertheless, as the clinical classification of SMA was established in the 1980s and the gene locus for 5q SMA was identified in the 1990s, the data and reviewed studies are related to 5q SMA and provide an overview of the natural history evidence that is available.

The effects of SMA treatment have been reported to vary depending on the clinical outcome assessed; for example, DMTs have been demonstrated to improve motor function in patients with SMA [23, 70–73]; however, effects on bulbar function are not as evident [74–76].

The heterogeneity of the results presented emphasizes that there are no standardized assessments used in clinical practice to record the bulbar symptoms of SMA. Therefore, generalizations of treatment effects across SMA symptoms cannot be made. This, in addition to the clinical consequences of bulbar function decline in most patients with SMA who do not receive DMT, highlights the importance of monitoring respiratory and bulbar function outcomes.

## CONCLUSIONS

Natural history data remain an important comparator in the assessment and acceptance of new treatments. This SLR provides a comprehensive repository of natural history data on respiratory and bulbar function in patients with Types 1–3 SMA. Findings indicated that greater declines in respiratory function were observed in patients with more severe disease. Respiratory function rapidly declined in patients with Type 1 SMA, with patients requiring respiratory support by 12 months of age. Patients with Type 1 SMA typically required NIV at an earlier age than those with Type 2 or 3 SMA who initiated respiratory support between the first and fifth decades of life.

Difficulties associated with swallowing, feeding, and speech were reported in patients with Types 1–3 SMA; however, their frequency and the types of deficits reported are determined by SMA type and the extent of SMA progression. A greater proportion of individuals with Type 1 SMA reported fatigue or shortness of breath when speaking than patients with Type 2 or 3 SMA. Individuals with Type 1 SMA also had the highest need for nutritional and feeding support.

The heterogeneity of swallowing outcomes identified in this SLR highlights that there are no standardized assessments routinely reported that measure bulbar function in SMA. In addition, the assessments that are available are not consistently applied across patients, making it difficult to assess and compare how bulbar function changes over the long term. Standardized evaluation of swallowing, feeding, and speech are needed to further understand the natural history of SMA.

## Supplementary Material

Supplementary Material

## Data Availability

Data from this study are available within the manuscript and supplementary material.
