# Supplementary material for: A Systematic Literature Review of the Natural History of Respiratory, Swallowing, Feeding, and Speech Functions in Spinal Muscular Atrophy (SMA)
Source: J Neuromuscul Dis. 2024 Sep 3;11(5):889–904. doi: 10.3233/JND-230248 (PMC11380303; doi:10.3233/JND-230248)
Supplement: Supplementary Material [file jnd-11-jnd230248-s001.pdf]

A Systematic Literature Review of the Natural History of Respiratory, Swallowing, Feeding, and Speech Functions in Spinal Muscular Atrophy (SMA)

Supplementary Table 1. Search strategy: Embase (Ovid)

**Search term**	**Results May 29th, 2021**	**Results June 27th, 2021**
spinal muscular atrophy/ or hereditary spinal muscular atrophy/	8,607	9,563
(spin$ adj3 musc$ adj3 atroph$).ti,ab,hw,ot.	11,520	12,750
((kennedy or kugelberg or werdnig hoffman$) adj2 (disease or syndrome)).ti,ab,hw.	2,992	3,257
or/1-3	12,596	13, 878
*prognosis/	53,341	58, 819
(prognos$ or predict$ or course or follow-up or episode$ or cohort or natural hist$).ti,ab.	5,673,278	6,161,855
(disease adj3 (progress$ or monitor$)).ti,ab.	289,090	318,076
exp longitudinal study/	156,199	174,235
or/5-8	5,902,044	6,411,667
exp scoliosis/su [Surgery]	8,438	9,143
exp artificial ventilation/ or exp assisted ventilation/	266,773	299,853
(bulbar adj2 function*).mp.	323	-
(bulbar adj2 function*).mp. [mp=title, abstract, heading word, drug trade name, original title, device manufacturer, drug manufacturer, device trade name, keyword heading word, floating subheading word, candidate term word]	-	357
(motor adj2 (function* or milestone*)).mp.	52,955	-
motor adj2 (function* or milestone*)).mp. [mp=title, abstract, heading word, drug trade name, original title, device manufacturer, drug manufacturer, device trade name, keyword heading word, floating subheading word, candidate term word]	-	57,911
speech/ or swallowing/	75,930	81,736
((respirat* or pulmonary or lung) adj2 (support or weak* or function*)).mp.	197,102	-
((respirat* or pulmonary or lung) adj2 (support or weak* or function*)).mp. [mp=title, abstract, heading word, drug trade name, original title, device manufacturer, drug manufacturer, device trade name, keyword heading word, floating subheading word, candidate term word]	-	210,685
scoliosis.mp.	36,663	39,340
(sit or sitting or walk* or ambulat* or stand*).mp.	2,884,965	-
(sit or sitting or walk* or ambulat* or stand*).mp. [mp=title, abstract, heading word, drug trade name, original title, device manufacturer, drug manufacturer, device trade name, keyword heading word, floating subheading word, candidate term word]	-	3,085,836

**Search term**	**Results May 29th, 2021**	**Results June 27th, 2021**
((feed* or nutrition* or speech or swallow*) adj (therap* or support* or difficult* or problem* or help or assist*)).mp.	66,974	-
((feed* or nutrition* or speech or swallow*) adj (therap* or support* or difficult* or problem* or help or assist*)).mp. [mp=title, abstract, heading word, drug trade name, original title, device manufacturer, drug manufacturer, device trade name, keyword heading word, floating subheading word, candidate term word]	-	71,854
exp contracture/su	3,521	3,670
contracture*.mp.	35,283	37,069
or/10-20	3,486,770	3,739,778
(accelerat* or change or progress* or rate or risk or loss or lost or lose or time or gain* or increas*).mp.	16,926,001	-
(accelerat* or change or progress* or rate or risk or loss or lost or lose or time or gain* or increas*).mp. [mp=title, abstract, heading word, drug trade name, original title, device manufacturer, drug manufacturer, device trade name, keyword heading word, floating subheading word, candidate term word]	-	18,144,859
21 and 22	2,092,018	2,273,346
9 or 23	7,317,660	7,936,580
4 and 24	4,063	4,695
limit 25 to yr="2021 -Current"	-	801

Searches were conducted from database inception (1974) to the search date.

Supplementary Table 2. Search strategy: Ovid MEDLINE and Epub Ahead of Print, In-Process, In-Data-Review and Other Non-Indexed Citations, MEDLINE Daily and Versions

**Search term**	**Results May 29th, 2021**	**Results June 27th, 2021**
exp Muscular Atrophy, Spinal/	5,366	6,037
(spin$ adj3 musc$ adj3 atroph$).ti,ab,hw,ot.	7,976	8,583
((kennedy or kugelberg or werdnig hoffman$) adj2 (disease or syndrome)).ti,ab,hw.	702	709
or/1-3	8,379	8,992
*Prognosis/	3,139	3,367
(prognos$ or predict$ or course or follow-up or episode$ or cohort or natural hist$).ti,ab.	3,984,247	4,338,841
(disease adj3 (progress$ or monitor$)).ti,ab.	171,918	188,964
exp Longitudinal Studies/	145,768	158,959
or/5-8	4,156,652	4,526,166
exp Scoliosis/su [Surgery]	8,030	8,750
exp Respiration, Artificial/	81,168	86,327
(bulbar adj2 function*).mp.	153	172
(motor adj2 (function* or milestone*)).mp.	34,716	37,879
Speech/	24,340	26,983
Deglutition/	10,142	11,025
((respirat* or pulmonary or lung) adj2 (support or weak* or function*)).mp.	118,140	125,055
scoliosis.mp.	26,479	28,010
(sit or sitting or walk* or ambulat* or stand*).mp.	2,424,154	2,578,043
((feed* or nutrition* or speech or swallow*) adj (therap* or support* or difficult* or problem* or help or assist*)).mp.	40,144	43,032
exp Contracture/su [Surgery]	4,074	4,233
contracture*.mp.	26,793	27,873
or/10-21	2,728,530	2,901,668
(accelerat* or change or progress* or rate or risk or loss or lost or lose or time or gain* or increas*).mp.	13,068,906	13,999,828
22 and 23	1,464,474	1,576,608
9 or 24	5,214,532	5,658,193
4 and 25	2,216	2,491
limit 26 to yr="2021 -Current"	-	408

Searches were conducted from database inception (1946) to search dates.

Supplementary Table 3. Search strategy: Evidence-Based Medicine Reviews (Ovid), Cochrane Central Register of Controlled Trials, and Cochrane Database of Systematic Reviews

**Search term**	**Results May 29th, 2021**	**Results June 27th, 2021**
exp Muscular Atrophy, Spinal/	96	103
(spin$ adj3 musc$ adj3 atroph$).ti,ab,hw,ot.	325	348
((kennedy or kugelberg or werdnig hoffman$) adj2 (disease or syndrome)).ti,ab,hw.	53	55
or/1-3	332	355
*Prognosis/	0	0
(prognos$ or predict$ or course or follow-up or episode$ or cohort or natural hist$).ti,ab.	436,668	448,988
(disease adj3 (progress$ or monitor$)).ti,ab.	27,740	27,616
exp Longitudinal Studies/	147,152	6,742
or/5-8	539,891	467,746
exp Scoliosis/su [Surgery]	14	0
exp Respiration, Artificial/	6,439	6,768
(bulbar adj2 function*).mp.	58	51
(motor adj2 (function* or milestone*)).mp.	8,263	8,232
Speech/	615	659
Deglutition/	418	469
((respirat* or pulmonary or lung) adj2 (support or weak* or function*)).mp.	27,504	27,682
scoliosis.mp.	1,644	1,615
(sit or sitting or walk* or ambulat* or stand*).mp.	308,722	321,810
((feed* or nutrition* or speech or swallow*) adj (therap* or support* or difficult* or problem* or help or assist*)).mp.	6,794	6,690
exp Contracture/su [Surgery]	40	2
contracture*.mp.	1,421	1,448
or/10-21	346,374	359,435
(accelerat* or change or progress* or rate or risk or loss or lost or lose or time or gain* or increas*).mp.	1,134,600	1,161,383
22 and 23	257,016	266,944
9 or 24	694,219	640,429
4 and 25	196	209
limit 26 to yr="2021 -Current"	-	29

Supplementary Table 4. Population, Intervention, Comparison, Outcomes, and Study framework used to define the research question and determine the eligibility criteria of this study

POPULATION	Patients with Types 1, 2, or 3 SMA; no restriction on phenotype
INTERVENTION	Not restricted by intervention
COMPARISON	Not relevant as part of the eligibility criteria as the effects of treatment on patient outcomes were not of interest for this systematic literature review
OUTCOMES	• Respiratory function/need for ventilatory support or tracheostomy (time-to-event data were of primary interest)<br>• Bulbar function (swallowing, feeding and speech); time-to-event data were of primary interest<br>• Respiratory outcomes:<br>– Type 2/3 SMA: FVC, PCF, and SNIP<br>– Type 1 SMA: Oximetry, polysomnography, blood gas analysis, and SNIP
STUDY DESIGN	• Prospective/retrospective observational/registry studies<br>• Case–control studies<br>• Cross-sectional surveys<br>• Prospective/retrospective case series

Abbreviations: FVC = forced vital capacity; PCF = peak cough flow; SMA = spinal muscular atrophy; SNIP = sniff nasal inspiratory pressure.

Supplementary Table 5. Studies included in the SLR that reported on respiratory and bulbar function outcomes (swallowing, feeding, and speech)

**Study**	**Title**
Alvarez 2019 [2]	Observations from a nationwide vigilance program in medical care for spinal muscular atrophy patients in Chile
Annoussamy 2021 [3]	Natural history of Type 2 and 3 spinal muscular atrophy: 2-year NatHis-SMA study
Aguerre 2020 [4]	Natural history of Type 1 spinal muscular atrophy in a series of Argentinian children
Berti 2021 [5]	Oral and Swallowing Abilities Tool (OrSAT) for Type 1 SMA patients: Development of a new module
Bladen 2014 [6]	Mapping the differences in care for 5,000 spinal muscular atrophy patients, a survey of 24 national registries in North America, Australasia and Europe
Brusa 2019 [7]	Secondary outcomes of spinal surgery in patients with spinal muscular atrophy (SMA)
Chabanon 2018 [8]	Prospective and longitudinal natural history study of patients with Type 2 and 3 spinal muscular atrophy: Baseline data NatHis-SMA study
Chen 2012 [9]	Prevalence and risk factors for feeding and swallowing difficulties in spinal muscular atrophy types II and III
Choi 2020 [10]	Trajectory of change in the swallowing status in spinal muscular atrophy type I
Darras 2022 [11]	Distribution of weight, stature, and growth status in children and adolescents with spinal muscular atrophy: an observational retrospective study in the United States
De Amicis 2021 [12]	Growth patterns in children with spinal muscular atrophy
De Sanctis 2016 [13]	Developmental milestones in type I spinal muscular atrophy
De Sanctis 2018 [14]	Clinical phenotypes and trajectories of disease progression in type 1 spinal muscular atrophy
Farrar 2013 [15]	Pathophysiological insights derived by natural history and motor function of spinal muscular atrophy
Finkel 2014 [16]	Observational study of spinal muscular atrophy type I and implications for clinical trials
Granata 1989 [17]	Spinal muscular atrophy: natural history and orthopaedic treatment of scoliosis
Johnson 2021 [18]	Characterization of adult patients with SMA treated in US hospital settings: a natural history study in the Premier Healthcare Database

Johnson 2022 [19]	POSC43 Healthcare resource use and activities of daily living status among adult patients with spinal muscular atrophy: a natural history multicountry chart review study
Kaneko 2017 [20]	Relationships between long-term observations of motor milestones and genotype analysis results in childhood-onset Japanese spinal muscular atrophy patients
Kapur 2019 [21]	Relationship between respiratory function and need for NIV in childhood SMA
Kaufmann 2012 [22]	Prospective cohort study of spinal muscular atrophy types 2 and 3
Mazzella 2022 [23]	Assessing perspectives of disease burden and clinically meaningful changes using the Spinal Muscular Atrophy Health Index in adolescents and young adults
McGrattan 2019 [24]	Natural history of physiologic swallowing deficits in spinal muscular atrophy type 1
Melemeni 2021 [25]	Respiratory function evaluation in treatment-naive patients with Spinal Muscular Atrophy
Messina 2008 [26]	Feeding problems and malnutrition in spinal muscular atrophy type II
Oskoui 2007 [27]	The changing natural history of spinal muscular atrophy type 1
Ou 2021 [28]	Natural history in spinal muscular atrophy Type I in Taiwanese population: a longitudinal study
Pane 2018 [29]	An observational study of functional abilities in infants, children, and adults with type 1 SMA
Sansone 2021 [30]	Sometimes they come back: new and old spinal muscular atrophy adults in the era of nusinersen
Seferian 2015 [31]	Upper limb evaluation and one-year follow up of non-ambulant patients with spinal muscular atrophy: an observational multicenter trial
Servais 2022 [32]	FIREFISH Parts 1 and 2: Safety and efficacy of risdiplam in Type 1 spinal muscular atrophy (3-year data)
Souchon 1996 [33]	Clinical and genetic study of chronic (types II and III) childhood onset spinal muscular atrophy
Trucco 2019 [34]	P.223 Respiratory function in SMA type 2 and non-ambulant SMA type 3, longitudinal data from the international SMA consortium (iSMAc)
Trucco 2020 [35]	Respiratory function in SMA type 2 and nonambulant SMA type 3: Longitudinal data from the international SMA consortium (iSMAc)

Trucco 2021 [36]	Respiratory Trajectories in Type 2 and 3 Spinal Muscular Atrophy in the iSMAC Cohort Study
van der Heul 2019 [37]	Bulbar Problems Self-Reported by Children and Adults with Spinal Muscular Atrophy
van der Heul 2020 [38]	Feeding and Swallowing Problems in Infants with Spinal Muscular Atrophy Type 1: An Observational Study
Veldhoen 2022 [39]	Natural history of respiratory muscle strength in spinal muscular atrophy: a prospective national cohort study
Wadman 2017 [40]	Association of motor milestones, SMN2 copy and outcome in spinal muscular atrophy types 0-4
Wadman 2021 [41]	Feeding difficulties in children and adolescents with spinal muscular atrophy type 2
Wijngaarde 2020 [42]	Natural history of lung function in spinal muscular atrophy
Wolfe 2020 [43]	Investigating temporal changes in percent predicted FVC and RULM score in non-Ambulant SMA type III children
Wolfe 2021 [44]	Longitudinal changes in respiratory and upper limb function in a pediatric type III spinal muscular atrophy cohort after loss of ambulation

Abbreviations: FVC = forced vital capacity; iSMAc = international Spinal Muscular Atrophy consortium; NatHis = natural history; NGT = nasogastric tube; NIV = non-invasive ventilation; RULM = Revised Upper Limb Module; SMA = spinal muscular atrophy; SLR = systematic literature review.

Supplementary Table 6. Studies excluded from the SLR based on full text analysis

Study	Title	Rationale for exclusion
**From search on May 29th, 2021 (*n* = 26)**		
Chacko 2021 [45]	Effect of nusinersen on respiratory function in paediatric spinal muscular atrophy Types 1-3	Outcome
Matsumoto 2021 [46]	Improvement of pulmonary function measured by patient-reported outcomes in patients with spinal muscular atrophy after growth-friendly instrumentation	Outcome
Otto 2021 [47]	Quantification of disease progression in spinal muscular atrophy with muscle MRI-a pilot study	Outcome
Pane 2021 [48]	Type I SMA new natural history: long-term data in nusinersen-treated patients	Outcome
Bartoli 2020 [49]	Outcomes in patients with spinal muscular atrophy given nusinersen, onasemnogene abeparvovec or no treatment: an analysis based on restricted mean survival time	Outcome
Ge 2020 [50]	[Effect of comprehensive health management on the prognosis of children with type I spinal muscular atrophy]	Non-English language
Mercuri 2020 [51]	Longitudinal natural history of type I spinal muscular atrophy: A critical review	Review/editorial
Hagenacker 2020 [52]	Nusinersen in adults with 5q spinal muscular atrophy: A non-interventional, multicentre, observational cohort study	Outcome
Shen 2020 [53]	Predictors for deformity progression in a spinal muscular atrophy cohort after scoliosis correction surgery	Outcome
Stolte 2020 [54]	Minimal clinically important differences in functional motor scores in adults with spinal muscular atrophy	Outcome
Paracha 2020 [55]	Pro62 Spinal muscular atrophy: Development of natural history models for disease subtypes	Outcome
Al-Zaidy 2019 [56]	Health outcomes in spinal muscular atrophy type 1 following AVXS-101 gene replacement therapy	Study design
Paradis 2019 [57]	Pro23 Healthcare resource utilization over three years among later childhood, adolescent and adult spinal muscular atrophy patients: A natural history study within U.S. hospitals	Outcome
Yamamoto 2018 [58]	[Pulmonary rehabilitation for patients with spinal muscular atrophy type II]	Non-English language
Belter 2018 [59]	An overview of the Cure SMA membership database: Highlights of key demographic and clinical characteristics of SMA members	Outcome

Bertini 2018 [60]	A prospective natural history study of type 1 spinal muscular atrophy	Study design
Darras 2018 [61]	Precious SMA natural history data: A benchmark to measure future treatment successes	Review/editorial
Wasserman 2017 [62]	Low bone mineral density and fractures are highly prevalent in pediatric patients with spinal muscular atrophy regardless of disease severity	Outcome
Holt 2017 [63]	Outcomes of primary posterior spinal fusion for scoliosis in spinal muscular atrophy: Clinical, radiographic, and pulmonary outcomes and complications	Outcome
Fujak 2013 [64]	Natural course of scoliosis in proximal spinal muscular atrophy type II and IIIa: descriptive clinical study with retrospective data collection of 126 patients	Outcome
Kaufmann 2011 [65]	Observational study of spinal muscular atrophy type 2 and 3: functional outcomes over 1 year	Linked publication
Deymeer 2008 [66]	Natural history of SMA IIIb: muscle strength decreases in a predictable sequence and magnitude	Outcome
Piepers 2008 [67]	A natural history study of late onset spinal muscular atrophy Types 3b and 4	Outcome
Swoboda 2005 [68]	Natural history of denervation in SMA: relation to age, *SMN2* copy number, and function	Outcome
Ignatius 1994 [69]	The natural history of severe spinal muscular atrophy--further evidence for clinical subtypes	Outcome
Thomas 1994 [70]	The natural history of type I (severe) spinal muscular atrophy	Outcome
**From search on June 27th, 2022 (*n* = 10)**		
Berti 2021 [5]	Oral and Swallowing Abilities Tool (OrSAT) for Type 1 SMA Patients: Development of a New Module [Duplicate publication]	Duplicate
Lemoine 2021 [71]	[The SMA France national registry: already encouraging results]	Outcome
Kong Kam Wa 2021 [72]	A case series of paediatric patients with spinal muscular atrophy type I undergoing scoliosis correction surgery	Population/disease not relevant
Duong 2021 [73]	A patient-centered evaluation of meaningful change on the 32-Item Motor Function Measure in spinal muscular atrophy using qualitative and quantitative data	Population/disease not relevant
Gonçalves 2021 [74]	Continuous noninvasive ventilatory support outcomes for patients with neuromuscular disease: a multicenter data collaboration	Population/disease not relevant

Chou 2021 [75]	Hybrid versus total sublaminar wires in patients with spinal muscular atrophy undergoing scoliosis surgery	Outcome
Gaume 2021 [76]	Minimally invasive fusionless surgery for scoliosis in spinal muscular atrophy: Long-term follow-up results in a series of 59 patients	Outcome
Stepien 2021 [77]	Motor function of children with SMA1 and SMA2 depends on the neck and trunk muscle strength, deformation of the spine, and the range of motion in the limb joints	Population/disease not relevant
Yang 2021 [78]	Natural history of spinal muscular atrophy in children: An analysis of 117 cases	Outcome
Alfano 2021 [79]	Validity and reliability of the neuromuscular gross motor outcome	Population/disease not relevant

Abbreviations: MRC = Medical Research Council; MRI = magnetic resonance image; SLR = systematic literature review; SMA = spinal muscular atrophy; SMN = survival of motor neuron.

Supplementary Table 7. Summary of studies reporting FVC outcomes

Author year	Data source, territory, population	N	Age	FVC outcome data reported
Announssamy 2021 [3]	NatHis-SMA<br><br>Belgium, France, and Germany<br><br>Types 2 and 3 SMA	81	Adults and children	•Median predicted FVC at 24 months: –2.63%; *p* = 0.012<br>•CFB FVC at 24 months: 0.02%; *p* = 0.10<br><br>Patients aged ≥6 years performed assessments whilst in the sitting position; the best results of three measurements were selected for analysis
Brusa 2019 [7] (abstract publication)	Patients who underwent scoliosis surgery at Great Ormand Street Hospital<br><br>UK<br><br>Types 2 and 3 SMA	33	Unclear	**Mean annual FVC decline, % (Type 2 SMA)**<br>•Pre-surgery: –7.4<br>•Post-surgery: –2.8 (*p* < 0.001 for pre- vs post-surgery)<br><br>Similar trajectories reported in patients with Type 3 SMA but data not reported<br><br>Methods of data collection were not reported in conference abstract publication
Chabanon 2018 [8]	NatHis-SMA<br><br>Belgium, France, and Germany<br><br>Types 2 and 3 SMA	81	Adults and children	**FVC % predicted values, median (IQR)**<br>•Type 2 SMA, non-sitter (*n* = 15): 44 (23–81)<br>•Type 2 SMA, sitter (*n* = 9): 62 (37–83)<br>•Type 3 SMA, non-ambulant (n = 9): 90 (77–105)<br>•Type 3 SMA, ambulant (*n* = 10): 96 (82–107)<br>•Overall (*n* = 43): 81 (43–92)<br><br>Patients >6 years of age performed FVC assessments in the sitting position which were captured with the Vitalograph spirometer; the best results of three measurements were selected for analysis
Granata 1989 [17]	Patients attending the Muscle Clinic of Rizzo Orthopaedic Institute between 1974 and 1988	63	Adults and children	•The absolute vital capacity corresponded with disease severity<br>•The absolute vital capacity among those losing the ability to walk varied wildly with an average value of 2600cc (range:

Author year	Data source, territory, population	N	Age	FVC outcome data reported
	Italy<br><br>Types 1–4 SMA			1,050–4,200) between the ages of 11 and 47 years<br>• Patients with intermediate SMA had a lower vital capacity averaging 1,150 cc (range: 700–1,900 cc) between the ages of 7 and 45 years, without a clear tendency to deteriorate with age<br><br>Methods of data collection were not reported in the publication
Kapur 2019 [21]	Children attending the Children's Health Queensland, Brisbane, Australia<br><br>Australia<br><br>Types 1–3 SMA	25	Children	**FVC % predicted, median (IQR) from a cross-sectional study:**<br>• Type 1 SMA: 57 (NR)<br>• Type 2 SMA: 51.25 (41.7)<br>• Type 3 SMA: 91.9 (12)<br>• All patients: 62 (53.2)<br><br>FVC Z scores:<br>• Children requiring NIV: –5.70<br>• Children not requiring NIV: –1.39 ($p \leq 0.02$)<br><br>Children who refused to perform a respiratory test were not forced. Standard testing methods were used
Kaufmann 2012 [22]	Three clinical sites of the Pediatric Neuromuscular Clinical Research Network for SMA<br><br>USA<br><br>Types 2 and 3 SMA	79	Children	**FVC % predicted at baseline, mean (SD)**<br>• Type 2 SMA: 45.6 (21.7)<br>• Type 3 SMA: 97.4 (17.5)<br>• All patients: 72.4 (32.6)<br><br>**Mean change in FVC over time was estimated using a repeated measures analysis of a covariance model, with time treated as a categorical variable shown graphically:**<br>• Mean change in FVC % predicted at 2 years: –3.14<br>• Mean change in FVC % predicted at 3 years: –2.92<br><br>The best results of three consecutive attempts for FVC using a Koko spirometer were selected for analysis
Melemeni 2021 [25]	Unclear data source and	20	Adults	**FVC % predicted at baseline, range:**

Author year	Data source, territory, population	N	Age	FVC outcome data reported
	territory (abstract publication)<br><br>Types 2–4 SMA			•Types 2–4 (*n* = 20): 20–87%<br><br>*“A progressive decline was observed in the respiratory parameters in SMA Type II whereas respiratory function was less affected in SMA Type III and IV”*
Sansone 2021 [30]	Patients from five Italian tertiary-care centers<br><br>Italy<br><br>Types 1–4 SMA	166	Adults	**FVC % predicted, mean (SD) for regularly followed-up patients:**<br>•Type 2 SMA, non-sitter (*n* = 17): 41.88 (29.26)<br>•Type 2 SMA, sitter (*n* = 18): 42.94 (20.09)<br>•Type 2 SMA, all (*n* = 35): 42.41 (24.72)<br>•Type 3 SMA, non-sitter (*n* = 4): 83.55 (21.13)<br>•Type 3 SMA, sitter (*n* = 28): 71.71 (31.96)<br>•Type 3 SMA, walker (*n* = 18): 87.78 (29.26)<br>•Type 3 SMA, all (*n* = 50): 78.44 (30.78)<br><br>**FVC % predicted, mean (SD) for newcomers:**<br>•Type 2 SMA, non-sitter (*n* = 14): 24.50 (20.69)<br>•Type 2 SMA, sitter (*n* = 2): 45.50 (0.70)<br>•Type 2 SMA, all (*n* = 16): 27.31 (20.56)<br>•Type 3 SMA, non-sitter (*n* = 3): 54.33 (9.61)<br>•Type 3 SMA, sitter (*n* = 26): 78.33 (20.83)<br>•Type 3 SMA, walker (*n* = 10): 102.44 (13.19)<br>•Type 3 SMA, all (*n* = 39): 82.15 (22.56)<br><br>Methods of data collection were not reported in the publication
Seferian 2015 [31]	Multicenter observational study of Upper Limb Evaluation in non-ambulant patients with a	23	Adults and children	**FVC % predicted at baseline, median (range)**<br>•Type 2 SMA (*n* = 11): 27 (16–70)<br>•Type 3 SMA (*n* = 5): 78 (48–100)

Author year	Data source, territory, population	N	Age	FVC outcome data reported
	neuromuscular disorder<br><br>France and Belgium<br><br>Types 2 and 3 SMA			
Souchon 1996 [33]	Followed in the Programme des maladies neuromusculaires at Hospital Marie Enfant in Montreal<br><br>Canada<br><br>Types 2 and 3 SMA	63	Adults and children	**FVC % at first visit, mean (SD)**<br>•Type 2 SMA: 54.9 (30.7)<br>•Type 3 SMA: 87 (26.3)<br>•All patients: 68.8 (32.8)<br><br>**FVC % at last visit, mean (SD)**<br>•Type 2 SMA: 37.4 (25.7)<br>•Type 3 SMA: 73.4 (29.9)<br>•All patients: 53.0 (32.7)<br><br>**FVC % decline % from first to last visit, mean (SD)**<br>•Type 2 SMA: –17.5 (20.4); p = 0.0001<br>•Type 3 SMA: –13.6 (17.5); p = 0.0012<br>•All patients: –15.8 (19.1); p = 0.0001<br><br>Follow-up duration 6.3 years (SD 2.9); FVC expressed as the percentage of the normal values expected for the patient's age and either height (in ambulant patients with no significant spinal deformities) or arm span (in other patients)
Trucco 2019* [34]	iSMAc centers (UK, USA, Italy)<br><br>UK, USA, Italy<br><br>Types 2 and 3 SMA (non-ambulant Type 3)	554	Children and adolescents	**FVC % predicted progression (available in n = 231):**<br>•Type 2 SMA: 3.3<br>•Type 3 SMA: 1.8<br><br>•In Type 2 SMA, FVC % predicted declined steeply from 5–15 years of age, followed by a leveling<br>•In Type 3 SMA, FVC % predicted declined slower but steadily from 10 years of age<br><br>Methods of data collection were not reported in the abstract publication

Author year	Data source, territory, population	N	Age	FVC outcome data reported
Trucco 2020* [35]	iSMAc centers (UK, USA, Italy)<br><br>UK, USA, Italy<br><br>Types 2 and 3 SMA (non-ambulant Type 3)	437	Children and adolescents	**Yearly rates of FVC % predicted progression (available in *n* = 260)**<br>• Type 2 SMA: 3.6<br>• Type 3 SMA: 3.5<br>• In Type 2 SMA, FVC % predicted declined steeply from 5–15 years of age, followed by a leveling<br>• In Type 3 SMA, FVC % predicted declined slower but steadily from 10 years of age<br><br>Methods of data collection were not reported in the abstract publication
Trucco 2021 [36]	iSMAc centers (UK, USA, Italy)<br><br>UK, USA, Italy<br><br>Types 2 and 3 SMA	437	Children	The decline in FVC % predicted in Type 2 SMA and Type 3 SMA followed different trajectories across age ranges (data displayed graphically)<br><br>• FVC % predicted decline from 5–13 years was 4.2% per year followed by a slower decline (1% per year) in Type 2 SMA<br>• FVC % predicted decline from 8–13 years was 6.3% per year followed by a slower decline (0.9% per year) in Type 3 SMA<br><br>Time to age at clinically meaningful thresholds of FVC (60, 40, and 20%) reported graphically<br>• Median age at FVC % predicted <60%: 12.8 years for Type 2 SMA<br>• Median age not reached for Type 3 SMA or for Type 2 or Type 3 SMA across the 40 and 20% FVC threshold<br><br>Spirometry was performed by either physiotherapists or respiratory physiologists who had received appropriate training in the context of clinical trials. FVC data were obtained from testing the patients in a sitting position. The best of three efforts deemed reliable by the operator was

Author year	Data source, territory, population	N	Age	FVC outcome data reported
				recorded according to international guidelines
Wijngaarde 2020 [42]	Patients enrolled in this study were participating in a prospective, population-based prevalence cohort study on SMA in The Netherlands (Wadman 2017, 2018, 2020)<br><br>The Netherlands<br><br>Types 1–4 SMA	170	Adults and children	Longitudinal predicted changes of FVC % displayed graphically with linear mixed model and non-linear model analyses of the longitudinal changes by SMA type<br><br>Annual rates of FVC % decline from fixed effect linear mixed-effect model:<br>• Type 1c SMA: 1.2%<br>• Type 2a SMA: 1.3%<br>• Type 2b SMA: 1.4%<br>• Type 3a SMA: 0.7%<br>• Type 3b SMA: 0.2%<br><br>FVC % predicted measurements were reported graphically using box plots to compare measurements taken in the sitting and supine positions<br><br>Annual rates of decline of FVC, graphical change in FVC over time, and FVC measurements in the sitting and supine position (all by SMA type)<br><br>Spirometry data were obtained from patients in an ongoing study using a handheld spirometer, and retrospective spirometry data were also included. All lung function tests were measured in a sitting position without corsets or braces and conducted using a small team of professionals experienced in lung function tests in patients with a neuromuscular disorder. Lung function tests in the supine position after a resting period were also conducted
Wolfe 2020 [43] (abstract publication superseded by Wolfe 2021)	Two UK centers<br><br>UK<br><br>Type 3 SMA (non-ambulant)	24	Children and adolescents	Wolfe 2020<br>• Median FVC % predicted score at baseline: 90%<br>• A significant progressive deterioration of 14.7% in FVC over the 24-month period was reported

Author year	Data source, territory, population	N	Age	FVC outcome data reported
Wolfe 2021 [44]				Methods of data collection were not reported in the abstract publication<br><br>Wolfe 2021<br>**FVC % predicted at baseline, median (range)**<br>•Type 3 SMA: 96 (66–131)<br><br>**FVC % predicted at 24 months, median (range)**<br>•Type 3 SMA: 80.5 (39–129)<br><br>**FVC % decline over 24 months, mean (SD)**<br>•Type 3 SMA: 17 (14.3); $p < 0.05$<br><br>Patient age showed a negative correlation with FVC % predicted score (shown graphically)

Abbreviations: CFB = change from baseline; FVC = forced vital capacity; IQR = interquartile range; iSMAc = international SMA consortium; NatHis = natural history; NIV = non-invasive ventilation; NR = not reported; RULM = Revised Upper Limb Module; SD = standard deviation; SMA = spinal muscular atrophy.
*Superseded by Trucco 2021 full publication.

Supplementary Fig. 1. PRISMA flow diagram for the original search (May 2021)

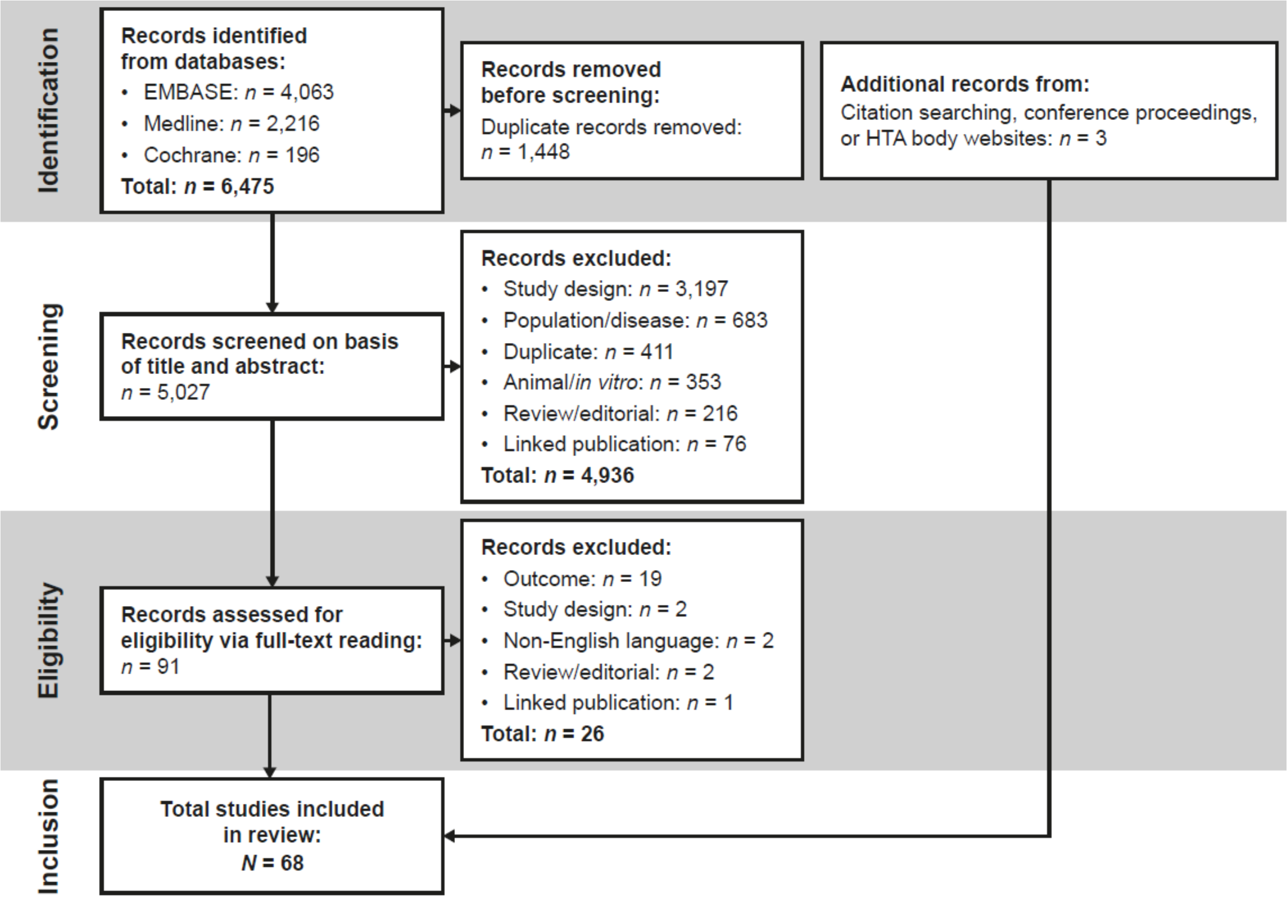

Figure modified from Aponte Ribero et al. 2023 [1].

HTA = health technology assessment; PRISMA = Preferred Reporting Items for Systematic Reviews and Meta-Analyses.

REFERENCES

1. Aponte Ribero V, Martí Y, Batson S, Mitchell S, Gorni K, Gusset N, et al. Systematic Literature Review of the Natural History of Spinal Muscular Atrophy: Motor Function, Scoliosis, and Contractures. Neurology. 2023:101(21):e2103-e2113. doi: 10.1212/WNL.0000000000207878.
2. Alvarez K, Suarez B, Palomino MA, Hervias C, Calcagno G, Martinez-Jalilie M, et al. Observations from a nationwide vigilance program in medical care for spinal muscular atrophy patients in Chile. Arq Neuropsiquiatr. 2019;77(7):470-477. doi: 10.1590/0004-282X20190073.
3. Annoussamy M, Seferian AM, Daron A, Pereon Y, Cances C, Vuillerot C, et al. Natural history of Type 2 and 3 spinal muscular atrophy: 2-year NatHis-SMA study. Ann Clin Transl Neurol. 2021;8(2):359-373. doi: 10.1002/acn3.51281.
4. Aguerre V, De Castro F, Mozzoni J, Gravina LP, Araoz HV, Monges S. Natural History of Type 1 Spinal Muscular Atrophy in a Series of Argentinian Children. J Neuromuscul Dis. 2020;7(4):453-458. doi: 10.3233/JND-200508.
5. Berti B, Fanelli L, de Sanctis R, Onesimo R, Palermo C, Leone D, et al. Oral and Swallowing Abilities Tool (OrSAT) for Type 1 SMA Patients: Development of a New Module. J Neuromuscul Dis. 2021;8(4):589-601. doi: 10.3233/JND-200614.
6. Bladen CL, Thompson R, Jackson JM, Garland C, Wegel C, Ambrosini A, et al. Mapping the differences in care for 5,000 spinal muscular atrophy patients, a survey of 24 national registries in North America, Australasia and Europe. J Neurol. 2014;261(1):152-163. doi: 10.1007/s00415-013-7154-1.
7. Brusa C, De Graaf J, Manzur A, Main M, Milev E, Iodice M, et al. 94 Secondary outcomes of spinal surgery in patients with spinal muscular atrophy (SMA): a retrospective analysis and a family-centred survey. Arch Dis Child. 2019;104:A37.
8. Chabanon A, Seferian AM, Daron A, Pereon Y, Cances C, Vuillerot C, et al. Prospective and longitudinal natural history study of patients with Type 2 and 3 spinal muscular atrophy: Baseline data NatHis-SMA study. PLoS One. 2018;13(7):e0201004. doi: 10.1371/journal.pone.0201004.
9. Chen YS, Shih HH, Chen TH, Kuo CH, Jong YJ. Prevalence and risk factors for feeding and swallowing difficulties in spinal muscular atrophy types II and III. J Pediatr. 2012;160(3):447-451.e441. doi: 10.1016/j.jpeds.2011.08.016.
10. Choi YA, Suh DI, Chae JH, Shin HI. Trajectory of change in the swallowing status in spinal muscular atrophy type I. Int J Pediatr Otorhinolaryngol. 2020;130:109818. doi: 10.1016/j.ijporl.2019.109818.
11. Darras BT, Guye S, Hoffart J, Schneider S, Gravestock I, Gorni K, et al. Distribution of weight, stature, and growth status in children and adolescents with spinal muscular atrophy: An observational retrospective study in the United States. Muscle Nerve. 2022;66(1):84-90. doi: 10.1002/mus.27556.
12. De Amicis R, Baranello G, Foppiani A, Leone A, Battezzati A, Bedogni G, et al. Growth patterns in children with spinal muscular atrophy. Orphanet J Rare Dis. 2021;16(1):375. doi: 10.1186/s13023-021-02015-9.
13. De Sanctis R, Coratti G, Pasternak A, Montes J, Pane M, Mazzone ES, et al. Developmental milestones in type I spinal muscular atrophy. Neuromuscul Disord. 2016;26(11):754-759. doi: 10.1016/j.nmd.2016.10.002.
14. De Sanctis R, Pane M, Coratti G, Palermo C, Leone D, Pera MC, et al. Clinical phenotypes and trajectories of disease progression in type 1 spinal muscular atrophy. Neuromuscul Disord. 2018;28(1):24-28. doi: 10.1016/j.nmd.2017.09.015.
15. Farrar MA, Vucic S, Johnston HM, du Sart D, Kiernan MC. Pathophysiological insights derived by natural history and motor function of spinal muscular atrophy. J Pediatr. 2013;162(1):155-159. doi: 10.1016/j.jpeds.2012.05.067.

16. Finkel RS, McDermott MP, Kaufmann P, Darras BT, Chung WK, Sproule DM, et al. Observational study of spinal muscular atrophy type I and implications for clinical trials. Neurology. 2014;83(9):810-817. doi: 10.1212/WNL.0000000000000741.
17. Granata C, Merlini L, Magni E, Marini ML, Stagni SB. Spinal muscular atrophy: natural history and orthopaedic treatment of scoliosis. Spine. 1989;14(7):760-762. doi: 10.1097/00007632-198907000-00019.
18. Johnson NB, Proud C, Wassel CL, Dreyfus J, Cochrane T, Paradis AD. Characterization of Adult Patients With SMA Treated in US Hospital Settings: A Natural History Study in the Premier Healthcare Database. J Neuromuscul Dis. 2021;02:02. doi: 10.3233/JND-200624.
19. Johnson N, Paradis AD, Dave V, Macahilig C, Johnson C, Stephens JM, et al. POSC43 Healthcare Resource Use and Activities of Daily Living Status Among Adult Patients with Spinal Muscular Atrophy: A Natural History Multicountry Chart Review Study. Value Health. 2022;25(1):S94.
20. Kaneko K, Arakawa R, Urano M, Aoki R, Saito K. Relationships between long-term observations of motor milestones and genotype analysis results in childhood-onset Japanese spinal muscular atrophy patients. Brain Dev. 2017;39(9):763-773. doi: 10.1016/j.braindev.2017.04.018.
21. Kapur N, Deegan S, Parakh A, Gauld L. Relationship between respiratory function and need for NIV in childhood SMA. Pediatr Pulmonol. 2019;54(11):1774-1780. doi: 10.1002/ppul.24455.
22. Kaufmann P, McDermott MP, Darras BT, Finkel RS, Sproule DM, Kang PB, et al. Prospective cohort study of spinal muscular atrophy types 2 and 3. Neurology. 2012;79(18):1889-1897. doi: 10.1212/WNL.0b013e318271f7e4.
23. Mazzella A, Cruz R, Belter L, Curry M, Dilek N, Zizzi C, et al. Assessing perspectives of disease burden and clinically meaningful changes using the Spinal Muscular Atrophy Health Index in adolescents and young adults. Muscle Nerve. 2022;66(3):276-281. doi: 10.1002/mus.27644.
24. McGrattan KE, Keeley M, McGhee H, Clemmens C, Hernandez K. Natural history of physiologic swallowing deficits in spinal muscular atrophy type 1. Dysphagia. 2019;34 (6):1004.
25. Melemeni A, Tsaroucha A, Papagiannakis N, Chatziioannou A, Papadopoulos C, Kararizou E, et al. Respiratory function evaluation in treatment-naive patients with Spinal Muscular Atrophy. Eur J Neurol. 2021;28(Suppl. 1):833. doi: 10.1016/j.nmd.2008.02.008.
26. Messina S, Pane M, De Rose P, Vasta I, Sorleti D, Aloysius A, et al. Feeding problems and malnutrition in spinal muscular atrophy type II. Neuromuscul Disord. 2008;18(5):389-393. doi: 10.1016/j.nmd.2008.02.008.
27. Oskoui M, Levy G, Garland CJ, Gray JM, O'Hagen J, De Vivo DC, et al. The changing natural history of spinal muscular atrophy type 1. Neurology. 2007;69(20):1931-1936. doi: 10.1212/01.wnl.0000290830.40544.b9.
28. Ou SF, Ho CS, Lee WT, Lin KL, Jones CC, Jong YJ. Natural history in spinal muscular atrophy Type I in Taiwanese population: A longitudinal study. Brain Dev. 2021;43(1):127-134. doi: 10.1016/j.braindev.2020.07.012.
29. Pane M, Palermo C, Messina S, Sansone VA, Bruno C, Catteruccia M, et al. An observational study of functional abilities in infants, children, and adults with type 1 SMA. Neurology. 2018;91(8):e696-e703. doi: 10.1212/WNL.0000000000006050.
30. Sansone VA, Coratti G, Pera MC, Pane M, Messina S, Salmin F, et al. Sometimes they come back: New and old spinal muscular atrophy adults in the era of nusinersen. Eur J Neurol. 2021;28(2):602-608. doi: 10.1111/ene.14567.
31. Seferian AM, Moraux A, Canal A, Decostre V, Diebate O, Le Moing AG, et al. Upper limb evaluation and one-year follow up of non-ambulant patients with spinal muscular atrophy: an observational multicenter trial. PLoS One. 2015;10(4):e0121799. doi: 10.1371/journal.pone.0121799.
32. Servais L, Baranello G, Boespflug-Tanguy O, Day JW, Deconinck N, Klein A, et al. FIREFISH Parts 1 and 2: Safety and efficacy of risdiplam in Type 1 spinal muscular atrophy

(3-year data). Presented at the European Paediatric Neurology Society Congress. May 2022.
33. Souchon F, Simard LR, Lebrun S, Rochette C, Lambert J, Vanasse M. Clinical and genetic study of chronic (types II and III) childhood onset spinal muscular atrophy. Neuromuscul Disord. 1996;6(6):419-424. doi: 10.1016/s0960-8966(96)00379-3.
34. Trucco F, Ridout D, Finkel R, Mercuri E, Scoto M, Muntoni F, et al. P.223 Respiratory function in SMA type 2 and non-ambulant SMA type 3, longitudinal data from the international SMA consortium (iSMAc). Neuromuscul Disord. 2019;29 (Suppl. 1):S131-S2.
35. Trucco FT, Ridout DR, Finkel RF, Mercuri EM, Scoto MS, Muntoni FM. Respiratory function in SMA type 2 and nonambulant SMA type 3: Longitudinal data from the international SMA consortium (iSMAc). Dev Med Child Neurol. 2020;62 (Suppl. 1):67.
36. Trucco F, Ridout D, Scoto M, Coratti G, Main ML, Muni Lofra R, et al. Respiratory Trajectories in Type 2 and 3 Spinal Muscular Atrophy in the iSMAC Cohort Study. Neurology. 2021;96(4):e587-e599. doi: 10.1212/WNL.0000000000011051.
37. van der Heul AMB, Wijngaarde CA, Wadman RI, Asselman F, van den Aardweg MTA, Bartels B, et al. Bulbar Problems Self-Reported by Children and Adults with Spinal Muscular Atrophy. J Neuromuscul Dis. 2019;6(3):361-368. doi: 10.3233/JND-190379.
38. van Der Heul AMB, Cuppen I, Wadman RI, Asselman F, Schoenmakers MAGC, Van De Woude DR, et al. Feeding and Swallowing Problems in Infants with Spinal Muscular Atrophy Type 1: An Observational Study. J Neuromuscul Dis. 2020;7(3):323-330. doi: 10.3233/JND-190465.
39. Veldhoen ES, Wijngaarde CA, Hulzebos EHJ, Wösten-van Asperen RM, Wadman RI, van Eijk RPA, et al. Natural history of respiratory muscle strength in spinal muscular atrophy: a prospective national cohort study. Orphanet J Rare Dis. 2022;17(1):70. doi: 10.1186/s13023-022-02227-7.
40. Wadman RI, Stam M, Gijzen M, Lemmink HH, Snoeck IN, Wijngaarde CA, et al. Association of motor milestones, SMN2 copy and outcome in spinal muscular atrophy types 0-4. J Neurol Neurosurg Psychiatry. 2017;88(4):365-367. doi: 10.1136/jnnp-2016-314292.
41. Wadman RI, De Amicis R, Brusa C, Battezzati A, Bertoli S, Davis T, et al. Feeding difficulties in children and adolescents with spinal muscular atrophy type 2. Neuromuscul Disord. 2021;31(2):101-112. doi: 10.1016/j.nmd.2020.12.007.
42. Wijngaarde CA, Veldhoen ES, van Eijk RPA, Stam M, Otto LAM, Asselman FL, et al. Natural history of lung function in spinal muscular atrophy. Orphanet J Rare Dis. 2020;15(1):88. doi: 10.1186/s13023-020-01367-y.
43. Wolfe A, Scoto M, Muni Lofra R, Milev E, Rohwer A, Wake R, et al. 84. Investigating temporal changes in percent predicted FVC and RULM score in non-Ambulant SMA type III children. Arch Dis Child. 2020;105 (Suppl. 2):A29. doi: 10.1136/archdischild-2020-gosh.84.
44. Wolfe A, Scoto M, Milev E, Muni Lofra R, Abbott L, Wake R, et al. Longitudinal changes in respiratory and upper limb function in a pediatric type III spinal muscular atrophy cohort after loss of ambulation. Muscle Nerve. 2021;64(5):545-551. doi: 10.1002/mus.27404.
45. Chacko A, Sly PD, Ware RS, Begum N, Deegan S, Thomas N, et al. Effect of nusinersen on respiratory function in paediatric spinal muscular atrophy types 1-3. Thorax. 2022;77(1):40-6. doi: 10.1136/thoraxjnl-2020-216564.
46. Matsumoto H, Mueller J, Konigsberg M, Ball J, St Hilaire T, Pawelek J, et al. Improvement of Pulmonary Function Measured by Patient-reported Outcomes in Patients With Spinal Muscular Atrophy After Growth-friendly Instrumentation. J Pediatr Orthop. 2021;41(1):1-5. doi: 10.1097/BPO.0000000000001656.
47. Otto LAM, Froeling M, van Eijk RPA, Asselman FL, Wadman R, Cuppen I, et al. Quantification of disease progression in spinal muscular atrophy with muscle MRI-a pilot study. NMR Biomed. 2021;34(4):e4473. doi: 10.1002/nbm.4473.
48. Pane M, Coratti G, Sansone VA, Messina S, Catteruccia M, Bruno C, et al. Type I SMA "new natural history": long-term data in nusinersen-treated patients. Ann Clin Transl Neurol. 2021;8(3):548-57. doi: 10.1002/acn3.51276.
49. Bartoli L, Messori A. Outcomes in patients with spinal muscular atrophy given nusinersen, onasemnogene abeparvovec or no treatment: an analysis based on restricted

mean survival time. Expert Opinion on Orphan Drugs. 2020;8(8):303-7. doi: 10.1080/21678707.2020.1802719
50. Ge XS, Qu YJ, Peng XY, Chen Q, Jiao H, Song F. [Effect of comprehensive health management on the prognosis of children with type I spinal muscular atrophy]. Zhonghua Er Ke Za Zhi. 2020;58(5):398-402. doi: 10.3760/cma.j.cn112140-20191225-00832.
51. Mercuri E, Lucibello S, Perulli M, Coratti G, de Sanctis R, Pera MC, et al. Longitudinal natural history of type I spinal muscular atrophy: a critical review. Orphanet J Rare Dis. 2020;15(1):84. doi: 10.1186/s13023-020-01356-1.
52. Hagenacker T, Wurster CD, Günther R, Schreiber-Katz O, Osmanovic A, Petri S, et al. Nusinersen in adults with 5q spinal muscular atrophy: a non-interventional, multicentre, observational cohort study. Lancet Neurol. 2020;19(4):317-25. doi: 10.1016/S1474-4422(20)30037-5
53. Shen PC, Lu CC, Liang WC, Tien YC, Jong YJ, Lu YM, et al. Predictors for Deformity Progression in a Spinal Muscular Atrophy Cohort After Scoliosis Correction Surgery. Clin Spine Surg. 2020;33(8):E407-E414. doi: 10.1097/BSD.0000000000000989.
54. Stolte B, Bois JM, Bolz S, Kizina K, Totzeck A, Schlag M, et al. Minimal clinically important differences in functional motor scores in adults with spinal muscular atrophy. Eur J Neurol. 2020;27(12):2586-94. doi: 10.1111/ene.14472.
55. Paracha N, Gorni K, Hudson P. PRO62 Spinal Muscular Atrophy: Development of Natural History Models for Disease Subtypes. Presented at the International Society for Pharmacoeconomics and Outcomes Research Congress. May 2020.
56. Al-Zaidy S, Pickard AS, Kotha K, Alfano LN, Lowes L, Paul G, et al. Health outcomes in spinal muscular atrophy type 1 following AVXS-101 gene replacement therapy. Pediatr Pulmonol. 2019;54(2):179-185. doi: 10.1002/ppul.24203.
57. Paradis AD, Wassel CL, Dreyfus J, Reyna SP, Johnson NB, Jhaveri M. PRO23 Healthcare Resource Utilization Over Three Years Among Later Childhood, Adolescent and Adult Spinal Muscular Atrophy Patients: A Natural History Study Within U.S. Hospitals. Presented at the International Society for Pharmacoeconomics and Outcomes Research Congress. November 2019.
58. Yamamoto H, Saito T, Nagayama H, Okamoto K, Matsumura T, Inoue K. Pulmonary rehabilitation for patients with spinal muscular atrophy type II. NO TO HATTATSU. 2018;50(5):337-341. doi: 10.11251/ojjscn.50.337.
59. Belter L, Cook SF, Crawford TO, Jarecki J, Jones CC, Kissel JT, et al. An overview of the Cure SMA membership database: Highlights of key demographic and clinical characteristics of SMA members. J Neuromuscul Dis. 2018;5(2):167-76. doi: 10.3233/JND-170292.
60. Bertini E, Mercuri E. Motor neuron disease: A prospective natural history study of type 1 spinal muscular atrophy. Nat Rev Neurol. 2018;14(4):197-8. doi: 10.1038/nrneurol.2017.189.
61. Darras BT, De Vivo DC. Precious SMA natural history data: A benchmark to measure future treatment successes. Neurology. 2018;91(8):337-9. doi: 10.1212/WNL.0000000000006026.
62. Wasserman HM, Hornung LN, Stenger PJ, Rutter MM, Wong BL, Rybalsky I, et al. Low bone mineral density and fractures are highly prevalent in pediatric patients with spinal muscular atrophy regardless of disease severity. Neuromuscul Disord. 2017;27(4):331-7. doi: 10.1016/j.nmd.2017.01.019.
63. Holt JB, Dolan LA, Weinstein SL. Outcomes of Primary Posterior Spinal Fusion for Scoliosis in Spinal Muscular Atrophy: Clinical, Radiographic, and Pulmonary Outcomes and Complications. J Pediatr Orthop. 2017;37(8):e505-e11. doi: 10.1097/BPO.0000000000001049.
64. Fujak A, Raab W, Schuh A, Richter S, Forst R, Forst J. Natural course of scoliosis in proximal spinal muscular atrophy type II and IIIa: descriptive clinical study with retrospective data collection of 126 patients. BMC Musculoskelet Disord. 2013;14:283. doi: 10.1186/1471-2474-14-283..

65. Kaufmann P, McDermott MP, Darras BT, Finkel R, Kang P, Oskoui M, et al. Observational study of spinal muscular atrophy type 2 and 3: functional outcomes over 1 year. Arch Neurol. 2011;68(6):779-86. doi: 10.1001/archneurol.2010.373.
66. Deymeer F, Serdaroglu P, Parman Y, Poda M. Natural history of SMA IIIb: muscle strength decreases in a predictable sequence and magnitude. Neurology. 2008;71(9):644-9. doi: 10.1212/01.wnl.0000324623.89105.c4.
67. Piepers S, van den Berg LH, Brugman F, Scheffer H, Ruiterkamp-Versteeg M, van Engelen BG, et al. A natural history study of late onset spinal muscular atrophy types 3b and 4. J Neurol. 2008;255(9):1400-4. doi: 10.1007/s00415-008-0929-0.
68. Swoboda KJ, Prior TW, Scott CB, McNaught TP, Wride MC, Reyna SP, et al. Natural history of denervation in SMA: relation to age, SMN2 copy number, and function. Ann Neurol. 2005;57(5):704-12. doi: 10.1002/ana.20473.
69. Ignatius J. The natural history of severe spinal muscular atrophy--further evidence for clinical subtypes. Neuromuscul Disord. 1994;4(5-6):527-8. doi: 10.1016/0960-8966(94)90094-9.
70. Thomas NH, Dubowitz V. The natural history of type I (severe) spinal muscular atrophy. Neuromuscul Disord. 1994;4(5-6):497-502. doi: 10.1016/0960-8966(94)90090-6.
71. Lemoine M, Gomez M, Grimaldi L, Urtizberea JA, Quijano-Roy S. [The SMA France national registry: already encouraging results]. Med Sci (Paris). 2021;37 Hors série n° 1:25-9. doi: 10.1051/medsci/2021187.
72. Kong Kam Wa T, Holmes C, O'Brien K. A case series of paediatric patients with spinal muscular atrophy type I undergoing scoliosis correction surgery. Anaesth Rep. 2021;9(2):e12138. doi: 10.1002/anr3.12138.
73. Duong T, Staunton H, Braid J, Barriere A, Trzaskoma B, Gao L, et al. A Patient-Centered Evaluation of Meaningful Change on the 32-Item Motor Function Measure in Spinal Muscular Atrophy Using Qualitative and Quantitative Data. Front Neurol. 2021;12:770423. doi: 10.3389/fneur.2021.770423.
74. Gonçalves MR, Bach JR, Ishikawa Y, Saporito L, Winck JC. Continuous noninvasive ventilatory support outcomes for patients with neuromuscular disease: a multicenter data collaboration. Pulmonology. 2021;27(6):509-17. doi: 10.1016/j.pulmoe.2021.06.007.
75. Chou SH, Li WW, Lu CC, Lin KL, Lin SY, Shen PC, et al. Hybrid versus total sublaminar wires in patients with spinal muscular atrophy undergoing scoliosis surgery. BMC Musculoskelet Disord. 2021;22(1):867. doi: 10.1186/s12891-021-04737-0.
76. Gaume M, Saudeau E, Gomez-Garcia de la Banda M, Azzi-Salameh V, Mbieleu B, Verollet D, et al. Minimally Invasive Fusionless Surgery for Scoliosis in Spinal Muscular Atrophy: Long-term Follow-up Results in a Series of 59 Patients. J Pediatr Orthop. 2021;41(9):549-558. doi: 10.1097/BPO.0000000000001897.
77. Stępień A, Gajewska E, Rekowski W. Motor Function of Children with SMA1 and SMA2 Depends on the Neck and Trunk Muscle Strength, Deformation of the Spine, and the Range of Motion in the Limb Joints. Int J Environ Res Public Health. 2021;18(17). doi: 10.3390/ijerph18179134.
78. Yang YY, Yuan P, Li M, Jiang L, Hong SQ. Natural history of spinal muscular atrophy in children: an analysis of 117 cases. Zhongguo Dang Dai Er Ke Za Zhi. 2021;23(10):1038-43. doi: 10.7499/j.issn.1008-8830.2106025.
79. Alfano LN, Iammarino MA, Reash NF, Powers BR, Shannon K, Connolly AM, et al. Validity and Reliability of the Neuromuscular Gross Motor Outcome. Pediatr Neurol. 2021;122:21-6. doi: 10.1016/j.pediatrneurol.2021.05.021.
